# Serum biochemical and inflammatory biomarker profiles in severe preeclampsia and their clinical predictive value

**DOI:** 10.5937/jomb0-63709

**Published:** 2026-06-16

**Authors:** Fan Yang, Yanping Liu, Yanyan Liu

**Affiliations:** 1 Yichun Maternal and Child Health Hospital, Department of Nursing, Yichun, China

**Keywords:** preeclampsia, disease severity, biochemical indicators, inflammatory response, risk prediction, preeklampsija, težina bolesti, biohemijski indikatori, inflamatorni odgovor, predviđanje rizika

## Abstract

**Background:**

Preeclampsia is a pregnancy-specific hypertensive disorder characterized by systemic endothelial dysfunction, metabolic disturbances, and inflammatory activation. However, comprehensive biochemical and inflammatory biomarker profiles associated with disease severity, particularly severe preeclampsia, remain insufficiently defined. This study aimed to investigate serum biochemical and inflammatory markers in preeclampsia and to evaluate their clinical predictive value for severe disease.

**Methods:**

In this retrospective case-control study, pregnant women were classified into severe preeclampsia (n=30), mild preeclampsia (n=30), and normal pregnancy control groups (n=30). Serum biochemical parameters, including alanine aminotransferase, aspartate aminotransferase, lactate dehydrogenase (LDH), uric acid, creatinine, albumin, total bilirubin, triglycerides, and total cholesterol, were measured. Inflammatory markers, including C-reactive protein (CRP), interleukin-6 (IL-6), interleukin-8, tumor necrosis factor-a, procalcitonin, neutrophil-to-lymphocyte ratio, and platelet-to-lymphocyte ratio, were analyzed. Group comparisons, Spearman correlation analyses, multivariable logistic regression, and receiver operating characteristic (ROC) curve analyses were performed.

**Results:**

Both biochemical and inflammatory markers showed significant stepwise alterations across the three groups, with the most pronounced abnormalities observed in severe preeclampsia (P &lt; 0.05). LDH, uric acid, CRP, IL-6, and albumin were significantly associated with disease severity. Multivariable logistic regression identified elevated LDH, uric acid, CRP IL-6, and reduced albumin as independent predictors of severe preeclampsia. ROC analysis demonstrated that the combined biomarker model achieved good discriminative performance, with an area under the curve of 0.89 (95% CI: 0.81-0.96).

**Conclusions:**

Severe preeclampsia is characterized by distinct serum biochemical and inflammatory biomarker profiles. An integrated biomarker-based model may serve as a practical tool for early identification and risk stratification of severe preeclampsia.

## Abstract (sr)

**Uvod:**

Preeklampsija je hipertenzivni poremećaj specifican za trudnoću koji karakteriše sistemska endotelna disfunkcija, metabolicki poremećaji i inflamatorna aktivacija. Međutim, sveobuhvatni biohemijski i inflamatorni biomarkeri povezani sa težinom bolesti, posebno teške preeklampsije, ostaju nedovoljno definisani. Cilj ove studije bio je da se ispitaju serumski biohemijski i inflamatorni markeri kod preeklampsije i da se proceni njihova klinicka prediktivna vrednost za tešku bolest.

**Metode:**

U ovoj retrospektivnoj studiji slucaj-kontrola, trudnice su klasifikovane u grupe sa teškom preeklampsijom (n = 30), blagom preeklampsijom (n = 30) i kontrolnom grupom sa normalnom trudnoćom (n = 30). Mereni su serumski biohemijski parametri, ukljucujući alanin aminotransferazu, aspartat aminotransferazu, laktat dehidrogenazu (LDH), mokraćnu kiselinu, kreatinin, albumin, ukupni bilirubin, trigliceride i ukupni holesterol. Analizani su inflamatorni markeri, ukljucujući C-reaktivni protein (CRP), interleukin-6 (IL-6), interleukin-8, faktor tumorske nekroze-a, prokalcitonin, odnos neutrofila i limfocita i odnos trombocita i limfocita. Izvršena su poređenja grupa, Spirmanove korelacione analize, multivarijantna logisticka regresija i analize ROC krive (recover operating characteristic).

**Rezultati:**

I biohemijski i inflamatorni markeri pokazali su znacajne postepene promene u sve tri grupe, sa najizraženijim abnormalnostima primećenim kod teške preeklampsije (P &lt; 0,05). LDH, mokraćna kiselina, CRP IL-6 i albumin bili su znacajno povezani sa težinom bolesti. Multivarijantna logisticka regresija identifikovala je povišen LDH, mokraćnu kiselinu, CRP IL-6 i smanjeni albumin kao nezavisne prediktore teške preeklampsije. ROC analiza je pokazala da je kombinovani model biomarkera postigao dobre diskriminativne performanse, sa površinom ispod krive od 0,89 (95% CI: 0,81-0,96).

**Zaključak:**

Tešku preeklampsiju karakterišu različiti serumski biohemijski i inflamatorni biomarkerski profili. Integrisani model zasnovan na biomarkerima može poslužiti kao praktičan alat za ranu identifikaciju i stratifikaciju rizika od teške preeklampsije.

## Introduction

Preeclampsia is a pregnancy-specific hypertensive disorder that typically occurs after 20 weeks of gestation and remains a leading cause of maternal and perinatal morbidity and mortality worldwide [1][2]. The condition is clinically heterogeneous, ranging from mild forms to severe preeclampsia characterized by multi-organ dysfunction, including hepatic injury, renal impairment, metabolic disturbances, and systemic inflammation [3][4]. Despite advances in obstetric management, the early identification of patients at risk for severe disease progression remains a major clinical challenge [1][2].

Accumulating evidence suggests that preeclampsia is not merely a disorder of blood pressure regulation but a complex systemic disease involving endothelial dysfunction, altered metabolic homeostasis, and immune-inflammatory activation. Serum biochemical abnormalities, such as elevated liver enzymes, increased uric acid and creatinine levels, dyslipidemia, and reduced albumin, reflect organ involvement and metabolic stress during disease progression [5][6]. In parallel, inflammatory mediators - including proinflammatory cytokines and acute-phase reactants - have been implicated in the exaggerated maternal inflammatory response observed in preeclampsia [7][8]. These alterations collectively indicate that circulating biochemical and inflammatory markers may capture important molecular features of disease severity.

Previous studies have investigated individual biomarkers in preeclampsia; however, results have often been inconsistent, and many reports have focused on single parameters or limited patient populations [9]. Moreover, comparative analyses across different clinical severities - particularly between mild and severe preeclampsia - remain insufficient [10][11]. From the perspective of medical biochemistry, an integrated evaluation of routinely available serum biochemical indices together with inflammatory markers may provide a more comprehensive and clinically applicable characterization of disease-related molecular changes.

Importantly, there is growing interest in translating biomarker findings into predictive tools that can support clinical decision-making. Receiver operating characteristic analysis and multivariable logistic regression models allow the assessment of both individual and combined biomarker performance for disease prediction [12][13][14]. However, data regarding the combined predictive value of biochemical and inflammatory indicators for severe preeclampsia are still limited.

Therefore, the present retrospective case-control study aimed to systematically analyze serum biochemical and inflammatory biomarker profiles in patients with severe preeclampsia, mild preeclampsia, and normal pregnancies. Furthermore, we sought to evaluate the clinical predictive value of selected biomarkers and their combinations for identifying severe preeclampsia, thereby providing biochemical evidence to support risk stratification and early clinical intervention.

## Materials and methods

### Study design and participants

This retrospective case-control study was conducted at a tertiary medical center. Clinical and laboratory data were collected from pregnant women who delivered during the study period. Participants were classified into three groups according to established diagnostic criteria: severe preeclampsia, mild preeclampsia, and normal pregnancy controls. Each group included approximately 30 participants.

Preeclampsia was diagnosed according to international guidelines as new-onset hypertension (≥140/90 mmHg) after 20 weeks of gestation accompanied by proteinuria or end-organ dysfunction. Severe preeclampsia was defined by severe hypertension (≥160/110 mmHg) and/or evidence of significant organ involvement, including hepatic, renal, neurological, or hematological complications [15]. Women with normal blood pressure and no obstetric or medical complications served as controls.

Exclusion criteria included pre-existing chronic hypertension, renal or hepatic disease, diabetes mellitus, autoimmune disorders, acute or chronic infections, multiple gestations, malignancy, and incomplete clinical or laboratory data.

### Data collection

Demographic and clinical information, including maternal age, gestational age at delivery, and relevant obstetric data, were obtained from electronic medical records. Venous blood samples were collected after overnight fasting prior to delivery and processed according to standard laboratory procedures.

### Serum biochemical measurements

Serum biochemical parameters were measured using routine automated analyzers (Roche Cobas 8000, Roche Diagnostics, Mannheim, Germany) in the hospital's central laboratory. The assessed biochemical indices included alanine aminotransferase (ALT), aspartate aminotransferase (AST), lactate dehydrogenase (LDH), uric acid (UA), creatinine (Cr), albumin (ALB), total bilirubin (TBIL), triglycerides (TG), and total cholesterol (TC). All assays were performed following the manufacturers' instructions and internal quality-control procedures.

### Inflammatory marker assessment

Serum inflammatory markers, including C-reactive protein (CRP), interleukin-6 (IL-6), interleukin-8 (IL-8), tumor necrosis factor-a (TNF-a), and procalcitonin (PCT), were measured using standardized immunoassays. Hematological parameters were obtained from routine complete blood counts. The neutrophil-to-lymphocyte ratio (NLR) and platelet-to-lymphocyte ratio (PLR) were calculated as derived inflammatory indices.

### Statistical analysis

Statistical analyses were performed using standard statistical software. Continuous variables were assessed for normality using the Shapiro-Wilk test. Normally distributed data are presented as mean ± standard deviation, while non-normally distributed data are expressed as median (interquartile range). Comparisons among the three groups were conducted using one-way analysis of variance or the Kruskal-Wallis test, as appropriate. Categorical variables were compared using the chi-square test.

Correlation analyses were performed to evaluate associations between serum biomarkers and disease severity. Variables showing significant associations in univariate analyses were entered into multivariable logistic regression models to identify independent predictors of severe preeclampsia. Multicollinearity was assessed prior to model construction. Receiver operating characteristic (ROC) curve analysis was used to evaluate the predictive performance of individual biomarkers and combined models. The area under the ROC curve (AUC) with corresponding 95% confidence intervals was calculated. All included participants had complete datasets for the analyzed variables; therefore, no missing-data imputation was required. Variables demonstrating associations with severe preeclampsia at P < 0.10 in univariate analyses were considered candidate predictors and entered into multi-variable logistic regression models after assessment of multicollinearity using variance inflation factors. A two-sided *P* value < 0.05 was considered statistically significant.

## Results

### Baseline clinical characteristics

A total of approximately 90 pregnant women were included in the study, comprising patients with severe preeclampsia, mild preeclampsia, and normal pregnancy controls (approximately 30 per group). Baseline demographic and obstetric characteristics are summarized in Table 1. No significant differences were observed among the three groups in terms of maternal age or gestational age at delivery (*P* > 0.05), indicating comparability between groups.

**Table 1:** Baseline Characteristics of study participants.

Variable	Control (n=30)	Mild PE (n=30)	Severe PE (n = 30)	*P* value
Age (years)	30.4 ± 4.2	31.1 ± 4.6	30.8 ± 4.9	0.72
Gestational age (weeks)	38.2 ± 1.1	37.6 ± 1.3	36.9 ± 1.5	0.08

### Comparison of serum biochemical markers among groups

Significant differences in multiple serum biochemical parameters were observed among the three groups (Table 2). Compared with normal pregnancy controls, patients with preeclampsia - particularly those with severe disease - exhibited markedly elevated levels of ALT, AST, LDH, uric acid, and creatinine (*all P* < 0.05). Serum albumin levels were significantly reduced in severe preeclampsia compared with both mild preeclampsia and controls (*P* < 0.01).

**Table 2:** Serum biochemical parameters.

Marker	Control	Mild PE	Severe PE	*P*
CRP (mg/L)	3.2 (2.1-4.6)	6.9 (4.8-9.2)	12.4 (9.1-16.8)	<0.001
IL-6 (pg/mL)	6.1 ± 2.0	12.8 ± 4.3	21.6 ± 6.8	<0.001
IL-8 (pg/mL)	9.4 ± 3.1	16.2 ± 4.9	24.8 ± 7.2	<0.001
TNF-a (pg/mL)	8.7 ± 2.6	14.1 ± 3.8	20.3 ± 5.1	<0.001
PCT (ng/mL)	0.04 ± 0.02	0.09 ± 0.04	0.18 ± 0.07	<0.001
NLR	2.3 ± 0.6	3.6 ± 0.9	5.1 ± 1.2	<0.001
PLR	118 ± 34	162 ± 41	214 ± 56	<0.001

Lipid metabolism parameters also showed significant alterations. Triglyceride and total cholesterol levels were progressively increased from controls to mild preeclampsia and were highest in the severe preeclampsia group (*P* < 0.05). These findings indicate increasing metabolic and organ dysfunction with disease severity.

### Comparison of inflammatory markers among groups

Inflammatory biomarkers demonstrated pronounced differences across the three groups (Table 3). Serum levels of CRP IL-6, IL-8, TNF-a, and PCT were significantly elevated in patients with preeclampsia compared with controls, with the highest concentrations observed in severe preeclampsia (*all P* < 0.01).

**Table 3:** Inflammatory markers.

Marker	Control	Mild PE	Severe PE	P
ALT (U/L)	18.6 ± 5.2	28.4 ± 7.6	45.9 ± 12.1	<0.001
AST (U/L)	20.1 ± 4.8	30.2 ± 6.9	52.3 ± 14.5	<0.001
LDH (U/L)	176 ± 32	238 ± 45	362 ± 68	<0.001
UA (*μ*mol/L)	258 ± 41	328 ± 53	412 ± 67	<0.001
Cr (*μ*mol/L)	58 ± 9	71 ± 12	92 ± 18	<0.001
ALB (g/L)	39.6 ± 3.4	34.8 ± 3.9	29.7 ± 4.1	<0.001
TG (mmol/L)	2.1 ± 0.4	2.8 ± 0.5	3.4 ± 0.6	<0.001
TC (mmol/L)	4.6 ± 0.7	5.3 ± 0.8	6.1 ± 0.9	<0.001

Derived inflammatory indices, including NLR and PLR, were also significantly increased in preeclamptic patients, showing a stepwise elevation from normal pregnancy to mild and severe preeclampsia (*P* < 0.05). These results reflect an enhanced systemic inflammatory response associated with disease severity.

### Correlation between biomarkers and disease severity

Correlation analysis revealed that several biochemical and inflammatory markers were significantly associated with preeclampsia severity. LDH, uric acid, CRP IL-6, and NLR showed moderate to strong positive correlations with disease severity (Spearman *r* ranging from 0.42 to 0.61, *P* < 0.01), whereas serum albumin was negatively correlated (*r* = -0.48, *P* < 0.01).

### Logistic regression and predictive performance

Variables demonstrating significant associations in univariate analyses were entered into multivariable logistic regression models. Elevated LDH, uric acid, CRP and IL-6, along with decreased albumin levels, were identified as independent predictors of severe preeclampsia (Table 4).

**Table 4:** Multivariable logistic regression for severe preeclampsia.

Variable	OR	95% CI	*P*
LDH	1.012	1.006-1.019	<0.001
UA	1.008	1.003-1.014	0.002
ALB	0.86	0.78-0.94	0.001
CRP	1.21	1.08-1.36	0.002
IL-6	1.15	1.06-1.26	<0.001

**Figure 1:**
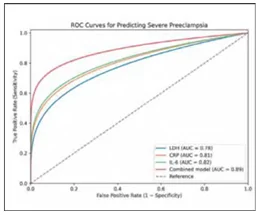
Baseline characteristics of study participants.

Receiver operating characteristic analysis demonstrated that individual biomarkers showed moderate predictive value for severe preeclampsia, with AUCs ranging from 0.70 to 0.82. Notably, a combined model incorporating LDH, uric acid, albumin, CRP and IL-6 achieved superior discriminative performance, with an AUC of 0.89 (95% CI: 0.81-0.96), indicating good predictive accuracy (Figure 1).

## Discussion

In the present retrospective case-control study, we systematically characterized serum biochemical and inflammatory biomarker profiles across normal pregnancy, mild preeclampsia, and severe preeclampsia. Our findings demonstrate that severe preeclampsia is associated with pronounced disturbances in liver and renal biochemical indices, lipid metabolism, and systemic inflammatory markers. Importantly, we further showed that a combined biomarker-based model integrating biochemical and inflammatory indicators provides good predictive performance for identifying severe preeclampsia.

One of the key observations of this study is the progressive alteration of serum biochemical markers with increasing disease severity. Elevated levels of ALT, AST, and LDH in severe preeclampsia reflect hepatocellular injury and widespread cellular stress, consistent with previous reports indicating hepatic involvement as a hallmark of severe disease. Increased uric acid and creatinine levels further suggest impaired renal function and altered purine metabolism, which are closely linked to endothelial dysfunction and oxidative stress. In contrast, the significant reduction in serum albumin observed in severe preeclampsia may reflect both decreased hepatic synthesis and increased vascular permeability, contributing to intravascular volume dysregulation. Collectively, these biochemical abnormalities underscore the multi-organ nature of severe preeclampsia and highlight their value as accessible indicators of disease burden.

In parallel, our results demonstrate a marked activation of systemic inflammation in preeclampsia, particularly in its severe form. Proinflammatory cytokines, including IL-6, IL-8, and TNF-a, were significantly elevated and exhibited stepwise increases from normal pregnancy to mild and severe preeclampsia [16][17][18]. These cytokines are known mediators of endothelial activation, vascular permeability, and immune dysregulation, which are central to the pathophysiology of preeclampsia. Elevated CRP and PCT levels further reflect an amplified acute-phase response, while increased NLR and PLR indicate a shift toward innate immune dominance. Together, these findings support the concept that severe preeclampsia represents a state of exaggerated inflammatory activation rather than a simple extension of mild disease.

Importantly, correlation analyses revealed that several biochemical and inflammatory markers were significantly associated with disease severity, reinforcing their potential clinical relevance. Among these, LDH, uric acid, CRP IL-6, and albumin showed particularly strong associations, suggesting that they capture complementary aspects of tissue injury, metabolic stress, and inflammation. Building on these observations, multivariable logistic regression identified a subset of these markers as independent predictors of severe preeclampsia. The combined predictive model demonstrated superior discriminative ability compared with individual biomarkers, as reflected by the ROC analysis. This finding highlights the advantage of integrating multiple biochemical dimensions rather than relying on single parameters.

From a medical biochemistry perspective, the strength of this study lies in its integrated approach. All included biomarkers are routinely measurable in clinical laboratories, making the proposed model potentially applicable in real-world settings. Unlike studies focusing on novel or experimental molecular markers [19][20], our findings emphasize the clinical value of conventional biochemical and inflammatory indices when interpreted collectively. Such an approach may facilitate early risk stratification, closer monitoring, and timely intervention for patients at risk of severe disease progression.

Nevertheless, several limitations should be acknowledged. First, the retrospective design may introduce selection bias and limits causal inference. Second, the relatively modest sample size may restrict statistical power and preclude extensive subgroup analyses. Potential confounding by gestational age at sampling and peripartum clinical interventions cannot be fully excluded. Although gestational age did not differ significantly between groups and blood samples were uniformly collected prior to delivery, inflammatory markers such as CRP and PCT may be influenced by subclinical inflammatory conditions. Patients with clinically evident infection were excluded; however, residual confounding remains possible and should be considered when interpreting the findings. In addition, although multicollinearity was assessed and the number of predictors was deliberately restricted to reduce the risk of overfitting, internal validation techniques such as bootstrapping or cross-validation were not performed due to sample size limitations. Finally, dynamic changes in biochemical and inflammatory biomarkers throughout pregnancy were not assessed, as only pre-delivery measurements were analyzed. Future prospective studies with larger cohorts and longitudinal sampling are warranted to validate, refine, and externally validate the proposed predictive model.

## Conclusion

In conclusion, severe preeclampsia is characterized by distinct and integrated alterations in serum biochemical and inflammatory markers. Combined biomarker-based models demonstrate promising predictive performance for severe disease and may serve as practical tools for clinical risk assessment. These findings provide biochemical evidence supporting the clinical utility of integrated serum markers in the management of preeclampsia.

## Dodatak

### Ethical considerations

This study was approved by the institutional ethics committee of Yichun Maternal and Child Health Hospital. Signed written informed consents were obtained from the patients and/or guardians. This study was conducted in accordance with the Declaration of Helsinki.

### Conflict of interest statement

All the authors declare that they have no conflict of interest in this work.
